# Individualised Multimodal Treatment Strategies for Anaplastic and Poorly Differentiated Thyroid Cancer

**DOI:** 10.3390/jcm7050115

**Published:** 2018-05-15

**Authors:** Sabine Wächter, Annette Wunderlich, Silvia Roth, Ioannis Mintziras, Elisabeth Maurer, Sebastian Hoffmann, Frederik A. Verburg, Sebastian A. Fellinger, Katharina Holzer, Detlef K. Bartsch, Pietro Di Fazio

**Affiliations:** 1 Department of Visceral, Thoracic and Vascular Surgery, Philipps University Marburg, Baldingerstrasse, D-35043 Marburg, Germany; seckhard@med.uni-marburg.de (S.W.); wunderli.an@gmail.com (A.W.); rothsi@med.uni-marburg.de (S.R.); ioannis.mintziras@uk-gm.de (I.M.); maurere@med.uni-marburg.de (E.M.); chirurgie@mkh-bgl.de (S.H.); katharina.holzer@uk-gm.de (K.H.); bartsch@med.uni-marburg.de (D.K.B.); 2 Department of Nuclear Medicine, Philipps University Marburg, Baldingerstrasse, D-35043 Marburg, Germany; frederikanton.verburg@uk-gm.de (F.A.V.); sebastian.fellinger@med.uni-marburg.de (S.A.F.)

**Keywords:** anaplastic thyroid cancer, poorly differentiated thyroid cancer, histone deacetylase inhibitors, sodium iodide symporter, *HMGA2*, miRNAs, epigenetics, tyrosine kinase inhibitors

## Abstract

The prognosis of anaplastic (ATC) and poorly differentiated thyroid cancer (PDTC) is poor, due to their radioiodine refractoriness (RAI-R), high metastatic potential and current lack of effective treatment strategies. We aimed to examine the efficacy of the tyrosine kinase inhibitors (TKIs) sorafenib and selumetinib and the histone deacetylase inhibitor (HDACI) panobinostat in patient-derived tumor tissue (PDTT) of ATCs/PDTCs, the expression of sodium iodide symporter (*NIS*) and radioiodine up-take (RAI-U). High Mobility Group AT-Hook 2 (*HMGA2*) and associated miRNAs expression was correlated with the clinical course of the patients. Inhibitory effects of panobinostat, sorafenib and selumetinib were measured by real time cell analyser xCELLigence in five PDTTs and human foreskin fibroblasts (HF) used as control. Expression of *NIS*, *HMGA2* and associated miRNAs hsa-let-7f-5p, hsa-let-7b-5p, hsa-miR-146b-5p and hsa-miR-146b-3p was performed by RT-qPCR and Western blot. RAI-U was performed by Gamma Counter with I-131. Panobinostat showed the strongest cytotoxic effect (10 nM) in all PDTTs and HF and caused a significant over-expression of *NIS* transcript. TKIs were able to up-regulate *NIS* transcript in patient 5 and in HF. RAI-U was up-regulated after 24 h of treatment with TKIs and panobinostat in all PDTT and HF, except in patient 5. Selumetinib caused a significant suppression of *HMGA2* in PDTT 1, 2, 4, 5 and HF; whereas sorafenib caused no change of *HMGA2* expression. Panobinostat suppressed significantly *HMGA2* in PDTT 2, 4 and HF. The expression of miRNAs hsa-let-7f-5p, has-let-7b-5p hsa-miR-146b-5p and hsa-miR-146b-3p was modulated heterogeneously. *NIS* protein level was over-expressed in three PDTTs (patients 1, 3 and 4) after 24 h of treatment with selumetinib, sorafenib and in particular with panobinostat. HF showed a stable *NIS* protein level after treatment. Panobinostat showed the strongest cytotoxicity in all treated PDTTs at the lowest dosage in comparison with TKI. All three compounds were able to modulate differently *NIS*, *HMGA2* and related miRNAs. These factors represent valuable markers in PDTT for new treatment strategies for patients suffering from ATC/PDTC. Thus, the establishment of PDTT could be a useful tool to test the efficacy of compounds and to develop new and individualised multimodal treatment options for PDTCs and ATCs.

## 1. Introduction

Although poorly differentiated thyroid carcinoma (PDTC) and anaplastic thyroid carcinoma (ATC) account only for a substantial portion of the morbidity and mortality associated with thyroid cancer (TC), both entities have unfavorable prognosis because of iodine/radioiodine refractoriness (RAI-R) and high metastatic potential.

Up to now, aggressive multimodal treatment based on surgical resection in combination with radio- and/or chemotherapy is recommended [1], but despite various attempts to improve the outcome of ATC and PDTC, the clinical course did not change significantly within the last decades [1,2].

The cellular origin of ATCs and PDTCs and the understanding of molecular mechanisms underlying thyroid carcinogenesis gained important implications for the therapeutic and follow-up strategies [3] and, in the last years, the tyrosine kinase inhibitors (TKIs) and histone deacetylase inhibitors (HDACIs) have shown promising results for the treatment of thyroid cancer [4].

Every individual ATC and PDTC has shown a different sensitivity to treatment with many different systemic compounds [5,6]. The resistance to the current available therapies is mainly due to the various epigenetic alterations occurring in these tumors; thus, playing a decisive role in tumorigenesis and metastasis, which characterise the aggressive clinical course of PDTCs and ATCs. Only a deeper understanding of the individual epigenetic dysregulations of individual ATCs and PDTCs may lead to the development of novel epigenetics-based therapeutic strategies [7]. Giordano et al. described the genomic landscape of 496 papillary thyroid carcinomas (PTCs) in 2014 and showed that the constitutive activation of the Mitogen-activated protein kinase (*MAPK*) cascade, mediated by oncogenic v-Raf murine sarcoma viral oncogene homolog B (*BRAF*) V600E mutation, down-regulates the expression of genes involved in iodine metabolism; thus resulting in RAI-R [8]. Nevertheless, the expression, modulation and function of the sodium iodide symporter (*NIS*; *SLC5A5*) in radioiodine refractory ATCs and PDTCs remain unclear. Some authors stated that the dysfunction of *NIS* is mainly due to suppression or loss of *NIS* gene [9]. Another mechanism causing dysfunction may be a distorted alignment at the cytosolic membrane site [10]. Furthermore, it has been shown that *NIS* is a target of miRNAs belonging to Let7 family. MiRNAs regulate gene expression by blocking translation or by inducing the degradation of their target mRNA. Damanakis et al. demonstrated the expression of *SLC5A5* (*NIS*) gene in ATCs and showed an inverse correlation between *SLC5A5* and the tumor suppressor microRNA (miRNA) hsa-let-7f-5p in thyroid cancer [11]. Hsa-let-7f-5p is inversely correlated with *NIS* expression due to its high affinity for the 3′UTR of *NIS* transcript [11,12,13]. De-regulation/suppression of Let-7 family members act in several types of cancer, including differentiated thyroid cancer (DTC) [14,15,16]. However, little is known regarding the distinct function of hsa-let-7f in TC. Among them, hsa-let-7f is described as critical for proper regulation, growth and differentiation of thyroid cells. In particular, hsa-let-7f-5p was reported to exert its tumor suppressor role by reducing cell proliferation and inducing thyroid differentiation markers [17]. Onco-miRNA hsa-miR-146b-5p is significantly over-expressed in papillary thyroid cancer (PTC) and associated with tumor migration, invasion and EMT (epithelial-mesenchymal transition) [12,18,19,20]. The over-expression of hsa-miR-146b-5p can be promoted by RET proto-oncogene (*RET/PTC3*) and *BRAF* activation [18].

High Mobility Group AT-Hook 2 (*HMGA2*), a well-known non-transcription factor with oncogenic properties, is highly expressed in several tumors [21,22], including TC [23]. It is responsible for supporting the activity of proliferation-related transcription factors, e.g., *E2F1*, leading to proliferation and survival of tumor cells [24].

*HMGA2* down-regulation, mediated by epigenetic modifications that imply the over-expression of Let-7 family miRNAs, leads to cell cycle block and cell death [25].

In this study, we aimed to examine the efficacy of two different TKIs, sorafenib and selumetinib and one HDACI, panobinostat, by using patient-derived tumor tissue (PDTT) of ATCs/PDTCs. We aimed to highlight the ability of these compounds to induce re-differentiation via the over-expression of *NIS* and associated radioiodine uptake (RAI-U). Furthermore, *HMGA2* and associated miRNAs expression were correlated with the clinical course of the patients.

## 2. Materials and Methods

### 2.1. Preparation of Patient-Derived Human Tumor Tissue (PDTT)

The tumor tissue resected from 5 patients who underwent surgery was immediately collected in sterile Phosphate-Buffer saline (PBS) without Ca^2+^ and Mg^+^ (L1825 Biochrom, Berlin, Germany). The tissue was washed (3×) with sterile PBS in order to remove any tissue debris and blood. Afterwards, the tissue was cut in small pieces with a sterile scalpel (Feather, Osaka, Japan). The small pieces were rinsed through a cell strainer (352350 BD Labware, Franklin Lakes, NJ, USA) and washed with Roswell Park Memorial Institute 1640 (RPMI1640) Medium (FG1215 Biochrom, Berlin, Germany). The cell suspension was centrifuged at 1500 rpm for 8 min at room temperature. The pellet was suspended with complete growth medium RPMI 1640 (Biochrom) supplemented with 10% fetal bovine serum (FBS, Biochrom) and 10 U/mL penicillin and 100 µg/mL streptomycin (Biochrom) and the suspension was pipetted in a cell culture 6-well plate (83.3920 Sarstedt, Nümbrecht, Germany). After 2 h, the adherence of the cells was checked. The medium was changed regularly every second day. The cells were then trypsinized and transferred into 25 cm^2^ flasks.

### 2.2. PDTT Cells and HF Culture Condition

All PDTT were grown in RPMI 1640 (Biochrom) supplemented with 10% fetal bovine serum (FBS, Biochrom) and 10 U/mL penicillin and 100 µg/mL streptomycin (Biochrom) under standard conditions (37 °C, 5% CO_2_). They were routinely tested for Mycoplasma contamination.

Human Foreskin Fibroblasts were grown in Dulbecco’s Modified Eagle Medium (DMEM) (Biochrom) supplemented with 10% fetal bovine serum (FBS, Biochrom) and 10 U/mL penicillin and 100 µg/mL streptomycin (Biochrom) under standard conditions (37 °C, 5% CO_2_). They were routinely tested for Mycoplasma contamination.

### 2.3. Compounds Tested

Selumetinib (AZD6244) was obtained from Selleck Chemicals (Houston, TX, USA). Sorafenib, *p*-Toluenesulfonate Salt was purchased from LC Laboratories (Woburn, MA, USA). Panobinostat was kindly provided from Novartis (Basel, Switzerland). All three compounds were dissolved in dimethylsulfoxide (DMSO) (WAK Chemicals; Steinbach, Germany) and stored at −20 °C.

### 2.4. Real-Time Cell Viability Analysis

The xCELLigence RTCA SP system (Roche Applied Science, Mannheim, Germany) was used for real-time and time-dependent analysis of the cellular response of human thyroid cancer cells following incubation with 1 to 100 µM selumetinib and sorafenib and 1 to 100 nM panobinostat. To perform this analysis, 3000 cells were seeded in 150 μL complete growth medium per well in a 96-well E-plate (OLS, Bremen, Germany) and incubated with the previously mentioned compounds. Cell index, which indicates attachment and adherence of cells to the plate’s electrode, was measured continuously for the following 130 h using the cell impedance detection system. Data analysis was performed using the RTCA Software v1.2.1 (OLS) for calculation of the temporal dynamics of cellular attachment (i.e., viability).

### 2.5. RNA Isolation and Quantitative Real-Time RT-PCR

Cells were seeded in 25 cm^2^ cell culture flasks (0.5 × 10^6^ cells/flask) and treated with 10 µM selumetinib, 10 µM sorafenib and 10 nM panobinostat for 48 h. Total RNA, including short RNA was isolated by use of miRNeasy Mini Kit (Qiagen, Hilden, Germany) according to the manufacturer’s instructions.

MiRNA enriched RNA was reverse transcribed with miScript II RT Kit (Qiagen). cDNA was amplified with miScript SYBR Green PCR Kit (Qiagen) using a CFX96 cycler (BioRad, Munich, Germany), hsa-let-7f-5p (MS00006489), hsa-let-7b-5p (MS00003122), hsa-miR-146b-5p (MS00003542) and hsa-miR-146b-3p (MS00008722) with miScript Primer Assays (Qiagen). RNU6 (MS00029204) was amplified as internal control miRNA.

For the amplification of *SLC5A5* and *HMGA2*, lysates were reverse transcribed using the iScript cDNA Syntesis Kit (Bio-Rad). PCR was run with the SsoFast Eva Green Supermix (BioRad) on CFX96 cycler. *GAPDH* was amplified as reference gene. Primer Assays for *SLC5A5* (PPH10926A) and *GAPDH* (QT01192646) were purchased from Qiagen. *HMGA2* (10025636) was purchased from BioRad.

Data Analysis: Results were analysed using CFX Manager (BioRad) and Rest 2008. Significance was calculated using the *t*-test for paired samples. *p* < 0.05 was regarded as significant.

### 2.6. Protein Isolation and Western Blotting

Cells were seeded in 75 cm^2^ cell culture flasks (1.5 × 10^6^ cells/flask) and incubated with 10 µM selumetinib, 10 µM sorafenib and 10 nM panobinostat for 48 h. The cell pellet was lysed with Radio Immuno Precipitation Assay (RIPA) (Santa Cruz, Heidelberg, Germany) Buffer containing protease and phosphatase inhibitors (71 µL 7 × protease cocktail and 50 µL 10× phosphatase cocktail (Roche, Basel, Switzerland) per 500 µL RIPA buffer). Protein content was determined by BCA-assay (Pierce, Rockford, LA, USA). Samples adjusted to 50 µg were separated on SDS-PAGE (NuPAGE Novex 4–12% Bis-Tris gels, NuPage MOPS running buffer (Invitrogen by Life Technologies, Carlsbad, CA, USA) and transferred to nitrocellulose (Amersham, Piscataway, NJ, USA). Membranes were probed using anti-human sodium iodide symporter (hNIS), clone FP5A (1:500) (Thermofisher Scientific, Fremont, CA, USA) as primary antibody. Horseradish Peroxidase (HRP) conjugated secondary antibodies were from Sigma-Aldrich. Visualization was performed by ECL western blotting reagent (Amersham) and using an image capture and analysis system (Fusion, PeqLab, Erlangen, Germany). Equal loading was verified by anti-GAPDH (Abcam 9485. 1:2500) (Abcam, Cambridge, MA, USA).

### 2.7. In Vitro Radioiodine Up-Take

For the analysis of radioiodine uptake (RAI-U), the cells were seeded in 6-well plates at a density of 4 × 10^5^ cells/well. Immediately after treatment with 10 µM selumetinib, 10 µM sorafenib and 10 nM panobinostat, 1 Mbq I-131 was added to the cells and the plates were incubated for 24 and 48 h. Subsequently, the cells were washed with PBS and trypsinized for 5 min. The suspension was rinsed with 3 mL PBS, collected and centrifuged at 900 rpm for 5 min. The supernatant was discarded and the pellet was suspended in 5 mL PBS. The suspension was once more centrifuged. The supernatant was discarded and the cells were processed into a Gamma counter for the measurement of the retained radioactive I-131. The untreated cells were used as control for the measurement.

### 2.8. Statistical Analysis

Data were collected using Excel (Microsoft Office 2010, Redmond, WA, USA). Significance was calculated using the *t*-test for paired samples. *p* < 0.05 was regarded as significant.

### 2.9. Ethical Approval

The study was conducted under the approval of the ethic committee of the University Hospital of Marburg (No. 92/15) and all the patients signed out the inform consent.

## 3. Results

Five PDTTs were established from five tumor tissues of patients affected by ATC and PDTC.

The patients were divided into two classes based on the tumor origin and the resected tissue. Primary tumor was collected from patients affected by ATC; lymph node metastases were resected from PDTC affected patients. Shown below are Table 1 for ATC patients and Table 2 for PDTC patients.

### 3.1. In Vitro Results of the First Patient

The first patient was affected by ATC and survived only two weeks after the first diagnosis. He received palliative treatment only and was characterised by BRAFV600 mutation and RAI-R (Table 1). No individualised therapy was established. The treatment with TKI and panobinostat was applied at the cells derived from his resected tumor after they have been kept proliferating in in vitro culture conditions.

#### 3.1.1. Effects on Cell Viability of the Individual Tumor Cells Caused by Treatment with Sorafenib, Panobinostat and Selumetinib

**Figure 1:**
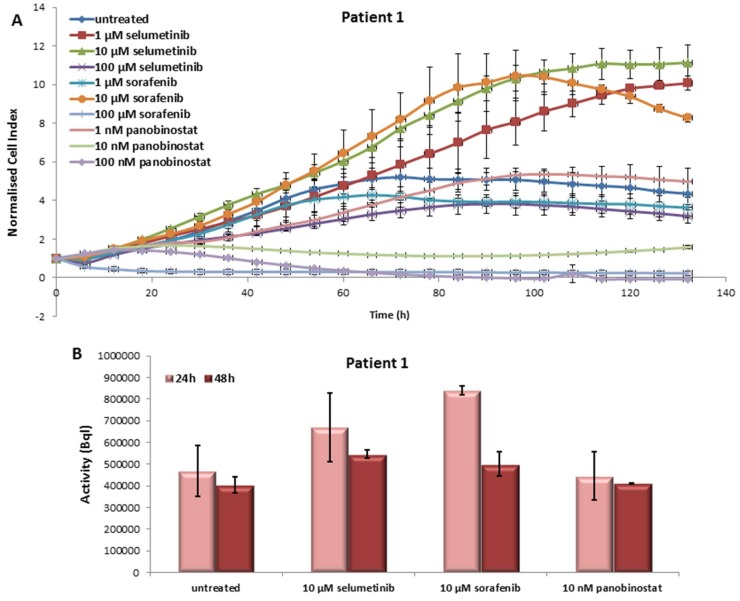
Effects of selumetinib, sorafenib and panobinostat on cell viability and RAI-U (radioiodine up-take). Real time cell viability of PDTT (patient-derived human tumor tissue) cells (anaplastic thyroid cancer—patient 1) treated with 1 to 100 µM selumetinib and sorafenib and 1 to 100 nM panobinostat (**A**). RAI-U after 24 and 48 h of treatment with 10 µM selumetinib, 10 µM sorafenib and 10 nM panobinostat (**B**). Shown are means of experiments performed in triplicates ± SD (**A**)/SEM (**B**).

Treatment with selumetinib showed no cytotoxicity in the PDTT isolated from this patient. Sorafenib caused a reduction of cell viability at 100 µM. Interestingly, panobinostat treatment caused a significant reduction of cell viability with 10 and 100 nM. The reduction of cell viability after prolonged treatment with 100 µM sorafenib and 10–100 nM panobinostat was nearly 100% (Figure 1A).

#### 3.1.2. Radioiodine Up-Take after Treatment with Sorafenib, Panobinostat and Selumetinib

The function of NIS protein was monitored by RAI-U measurement after administration of the three compounds. Radioiodine retention was up-regulated after 24 h of treatment with 10 µM selumetinib/10 µM sorafenib/10 nM panobinostat (Figure 1B). After 48 h of treatment, the cells of this PDTT showed no increase of RAI-U in comparison with the untreated cells.

#### 3.1.3. Expression of SLC5A5 (NIS), HMGA2 and miRNAs

**Figure 2:**
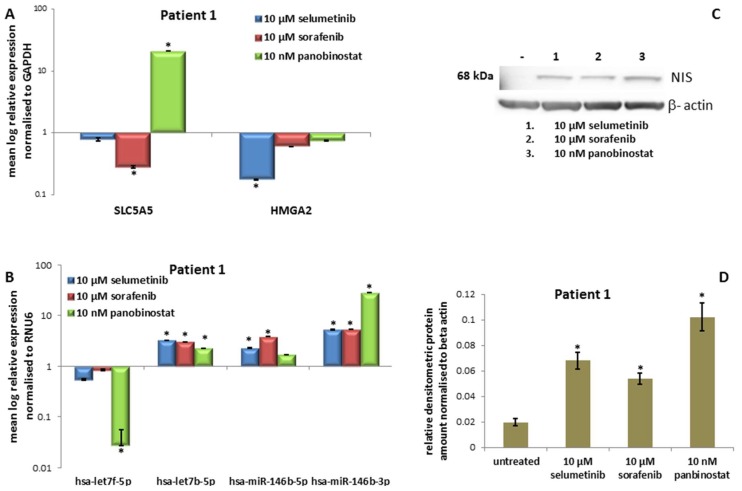
Effects of selumetinib, sorafenib and panobinostat on mRNAs, miRNAs and NIS protein expression in PDTT of patient 1 (ATC). RT-qPCR of *NIS*, *HMGA2* (**A**), hsa-let7f-5p, hsa-let7b-5p, hsa-miR-146b-5p and hsa-miR-146b-3p (**B**). The expression was normalised to glyceraldehyde 3-phosphate dehydrogenase (GAPDH) (**A**) and RNU6 (**B**) set at 1.0. Shown are means of experiments performed in triplicates ± SEM. * *p* < 0.05 regarded as significant. Western blot detection of NIS protein in PDTT cells treated with 10 µM selumetinib, 10 µM sorafenib and 10 nM panobinostat (**C**). Beta-actin was detected as quality loading control. (**D**) Densitometry of NIS protein level normalised to beta-actin. Shown are means of experiments performed in triplicates ± SEM. * *p* < 0.05 regarded as significant.

Treatment with 10 nM panobinostat induced a significant over-expression of SLC5A5 transcript, whereas 10 µM sorafenib determined a significant down-regulation of it and selumetinib did not cause any change of SLC5A5 expression (Figure 2A). HMGA2 transcript was significantly down-regulated after treatment with 10 µM selumetinib. The administration of sorafenib and panobinostat did not alter the expression of HMGA2 in comparison with untreated cells (Figure 2A).

Moreover, treatment with 10 nM panobinostat caused a significant down-regulation of hsa-let7f-5p (Figure 2B). After treatment with selumetinib and sorafenib, a significant up-regulated expression of hsa-let7b-5p, hsa-miR-146b-5p and hsa-miR-146b-3p was observed. Treatment with 10 nM panobinostat caused a significant over-expression of has-let7b-5p and hsa-miR-146b-3p.

#### 3.1.4. Expression of NIS Protein Level

Additionally, NIS protein level was analysed after 48 h of treatment with 10 µM selumetinib, 10 µM sorafenib and 10 nM panobinostat. As shown in Figure 2C, the untreated cells showed no detectable band corresponding to NIS protein. The treatment with the three compounds caused a strong significant re-expression of NIS protein as confirmed by densitometry analysis (Figure 2D).

Despite the TKIs were not showing a significant effect on the modulation of NIS transcript, the protein level was over-expressed after treatment.

Here, it could be observed that TKI were able to increase the radioiodine up-take and the protein level of NIS without any inverse correlation with has-let7f-5p. HMGA2 was significantly suppressed only after treatment with 10 µM selumetinib.

The higher potential of panobinostat in terms of benefit for patients affected by PDTC/ATC could be attributed to its low dosage and stronger cytotoxic effect that involve the suppression of the regulatory miRNA hsa-let7f-5p and the over-expression of NIS at transcript and protein level. The other miRNAs were over-expressed after treatment with TKI and panobinostat.

### 3.2. In Vitro Results of the Second Patient

The second patient was affected by ATC and survived for eight weeks after the first diagnosis. He received palliative treatment only and was characterized by RAI-R. No mutation of BRAF was detected (Table 1). He received palliative treatment and no individualized therapy was established. The treatment with TKIs and panobinostat was applied at the cells derived from his resected tumor after they have been kept proliferating in in vitro culture conditions.

#### 3.2.1. Effects on Cell Viability of the Individual Tumor Cells Caused by Treatment with Sorafenib, Panobinostat and Selumetinib

**Figure 3:**
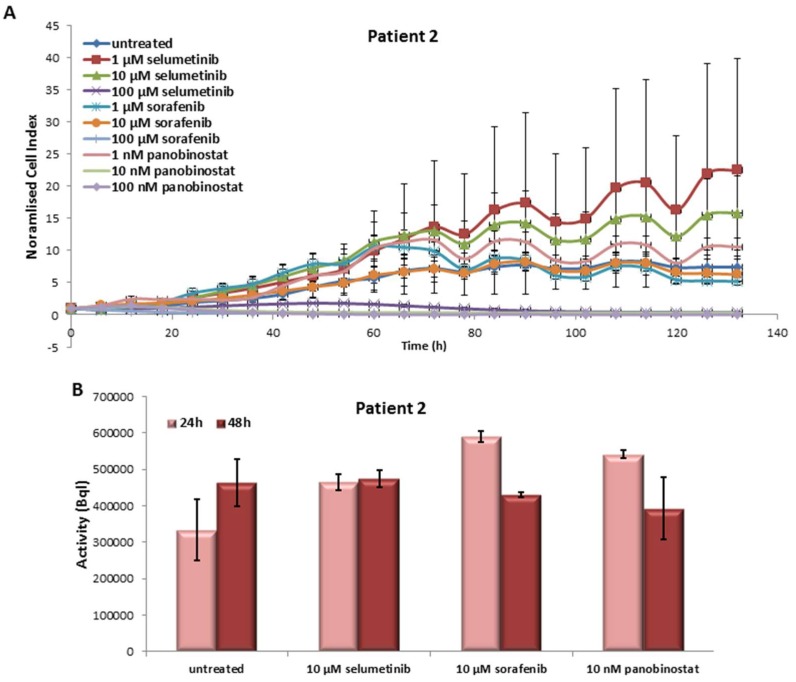
Effects of selumetinib, sorafenib and panobinostat on cell viability and RAI-U. Real time cell viability of PDTT (ATC—Patient 2) cells treated with 1 to 100 µM selumetinib and sorafenib and 1 to 100 nM panobinostat (**A**). RAI-U after 24 and 48 h of treatment with 10 µM selumetinib, 10 µM sorafenib and 10 nM panobinostat (**B**). Shown are means of experiments performed in triplicates ± SD (**A**)/SEM (**B**).

The PDTT obtained from the tissue of this patient showed a significant reduction of cell viability with 100 µM selumetinib and sorafenib (Figure 3A). Panobinostat showed a significant effect at 10 and 100 nM that determined a reduction of the growth curve to zero values.

#### 3.2.2. Radioiodine Up-Take after Treatment with Sorafenib, Panobinostat and Selumetinib

An increase of NIS monitored via RAI-U was observed after 24 h of treatment with 10 µM selumetinib, 10 µM sorafenib and 10 nM panobinostat (Figure 3B). After 48 h of treatment, the cells showed no change of RAI-U in comparison with untreated cells.

#### 3.2.3. Expression of *SLC5A5* (*NIS*), *HMGA2* and Their Regulatory miRNAs

**Figure 4:**
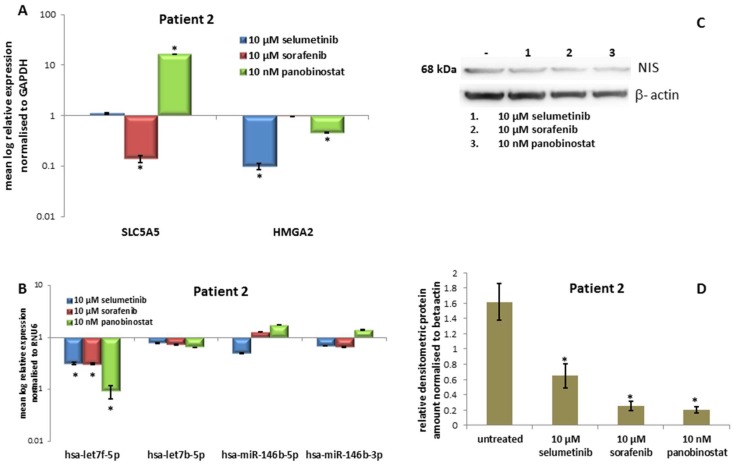
Effects of selumetinib, sorafenib and panobinostat on mRNAs, miRNAs and *NIS* protein expression in PDTT of patient 2 (ATC). RT-qPCR of *NIS*, *HMGA2* (**A**), hsa-let7f-5p, hsa-let7b-5p, hsa-miR-146b-5p and hsa-miR-146b-3p (**B**). The expression was normalised to GAPDH (**A**) and RNU6 (**B**) set at 1.0. Shown are means of experiments performed in triplicates ± SEM. * *p* < 0.05 regarded as significant. Western blot detection of *NIS* protein in PDTT cells treated with 10 µM selumetinib, 10 µM sorafenib and 10 nM panobinostat (**C**). Beta-actin was detected as quality loading control. (**D**) Densitometry of NIS protein level normalised to beta-actin. Shown are means of experiments performed in triplicates ± SEM. * *p* < 0.05 regarded as significant.

Here, treatment with 10 nM panobinostat induced a significant over-expression of *SLC5A5* transcript (Figure 4A). In addition, 10 µM sorafenib caused a significant down-regulation of *SLC5A5* transcript in PDTT. Furthermore, 10 µM selumetinib and 10 nM panobinostat caused a significant down-regulation of *HMGA2* transcript (Figure 4A). In this case, the treatment with selumetinib, sorafenib and especially panobinostat caused a down-regulation of hsa-let7f-5p (Figure 4B). Hsa-let7b-5p, hsa-miR-146b-5p and hsa-miR-146b-3p showed an almost stable expression after treatment with the three compounds.

#### 3.2.4. Expression of NIS Protein Level

The protein NIS was normally expressed in untreated cells. 48 h of treatment with 10 µM selumetinib, sorafenib and 10 nM panobinostat caused a significant reduction of NIS protein level as shown in Figure 4C, as confirmed by densitometry (Figure 4D).

Here, panobinostat showed the highest efficacy with the lowest dosage (10 and 100 nM). Furthermore, panobinostat alone caused an over-expression of *NIS* transcript together with the suppression of *HMGA2* and hsa-let7f-5p. 10 µM selumetinib was able to down-regulate *HMGA2* and hsa-let7f-5p. 10 µM sorafenib showed cytotoxic effects and the suppression of hsa-let7f-5p only. TKI and panobinostat increased the radioiodine up-take, whereas NIS protein level was down-regulated. The expression of the other miRNAs was stable after treatment with the three compounds.

### 3.3. In Vitro Results of the Third Patient

The third patient was affected by ATC and he is still alive after 282 weeks from his first diagnosis. After initial treatment with docetaxel and cisplatin, his therapy was interrupted and an individualised therapy with sorafenib was established. Treatment with sorafenib was characterised by multiple adverse effects and stopped. RAI-R was diagnosed. No mutation of BRAF was detected (Table 1). The treatment with TKI and panobinostat was applied at the cells derived from his resected tumor after they have been kept proliferating in in vitro culture conditions.

#### 3.3.1. Effects on Cell Viability of the Individual Tumor Cells Caused by Treatment with Sorafenib, Panobinostat and Selumetinib

**Figure 5:**
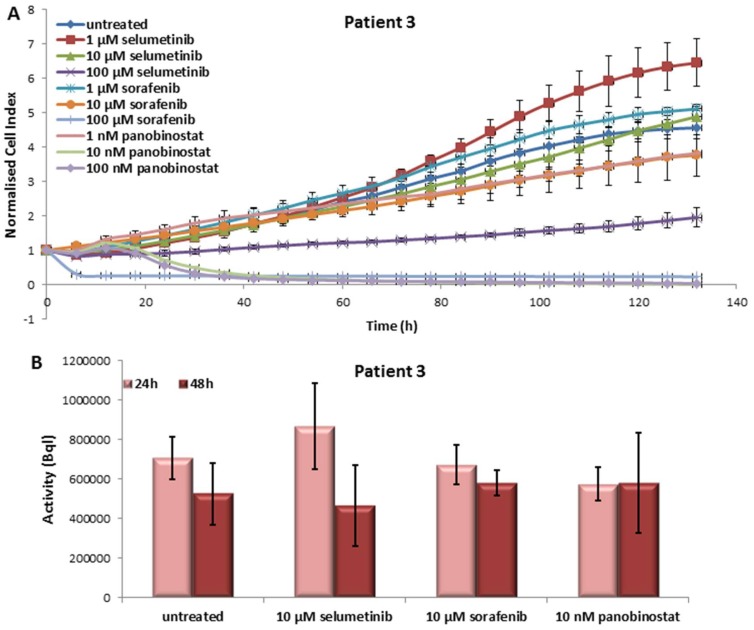
Effects of selumetinib, sorafenib and panobinostat on cell viability and RAI-U. Real time cell viability of PDTT cells (ATC—Patient 3) treated with 1 to 100 µM selumetinib and sorafenib and 1 to 100 nM panobinostat (**A**). RAI-U after 24 and 48 h of treatment with 10 µM selumetinib, 10 µM sorafenib and 10 nM panobinostat (**B**). Shown are means of experiments performed in triplicates ± SD (**A**)/SEM (**B**).

In this case, sorafenib and panobinostat were the most effective compounds. Treatment with 10–100 nM panobinostat and 100 µM sorafenib resulted in nearly 100% reduction of cell viability. As shown in Figure 5A, a significant reduction of cell viability could also be observed after treatment with 100 µM selumetinib.

#### 3.3.2. Radioiodine Up-Take after Treatment with Sorafenib, Panobinostat and Selumetinib

The activity of NIS, in terms of RAI-U, was only significantly up-regulated after 24 h of treatment with 10 µM selumetinib (Figure 5B). After 48 h of treatment, no increase of RAI-U was observed.

#### 3.3.3. Expression of *SLC5A5* (*NIS*), *HMGA2* and miRNAs

**Figure 6:**
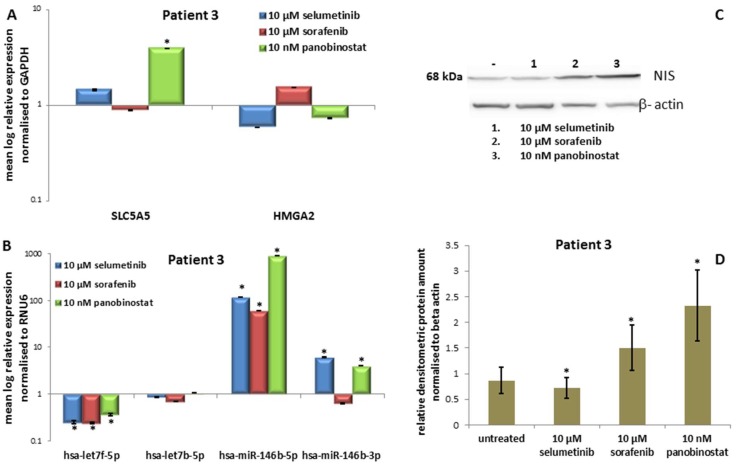
Effects of selumetinib, sorafenib and panobinostat on mRNAs, miRNAs and NIS protein expression in PDTT of patient 3 (ATC). RT-qPCR of *NIS*, *HMGA2* (**A**), hsa-let7f-5p, hsa-let7b-5p, hsa-miR-146b-5p and hsa-miR-146b-3p (**B**). The expression was normalised to GAPDH (**A**) and RNU6 (**B**) set at 1.0. Shown are means of experiments performed in triplicates ± SEM. * *p* < 0.05 regarded as significant. Western blot detection of NIS protein in PDTT cells treated with 10 µM selumetinib, 10 µM sorafenib and 10 nM panobinostat (**C**). Beta-actin was detected as quality loading control; (**D**) densitometry of NIS protein level normalised to beta-actin. Shown are means of experiments performed in triplicates ± SEM. * *p* < 0.05 regarded as significant.

Alone the treatment with 10 nM panobinostat induced a significant over-expression of *SLC5A5* (Figure 6A). Neither treatment with panobinostat nor selumetinib nor sorafenib caused a significant change of *HMGA2* transcript level.

Panobinostat, selumetinib and sorafenib caused a significant down-regulation of hsa-let7f-5p (Figure 6B). Hsa-let7b-5p was stably expressed in comparison with untreated cells. After treatment with every single compound, an up-regulation of hsa-miR-146b-5p was observed. Furthermore, selumetinib and panobinostat induced an over-expression of hsa-miR-146b-3p.

#### 3.3.4. Expression of NIS Protein Level

Treatment with 10 µM sorafenib and especially with 10 nM panobinostat was able to significantly increase the protein level of *NIS* as shown in Figure 6C; the treatment with 10 µM selumetinib caused no change of NIS protein. Figure 6D shows the densitometry analysis of NIS protein.

Once again, it could be observed the highest efficacy of panobinostat able to reduce cell viability at lower concentrations than TKI. Furthermore, panobinostat was able to up-regulate *NIS* transcript and protein level. This effect could be inversely correlated with the suppression of hsa-let7f-5p. In addition, 10 µM selumetinib caused an increase of RAI-U and a down-regulation of hsa-let7f-5p. The miRNAs mi-146b were over-expressed after treatment with the three single compounds.

### 3.4. In Vitro Results of the Fourth Patient

The fourth patient was affected by PDTC and the overall survival was 396 weeks after his first diagnosis. Radiotherapy was applied three times between 2010 and 2011. The patient was then treated with carboplatin and etoposide. The second line treatment was based on sorafenib and it was characterised by adverse events. RAI-R was diagnosed. No mutation of BRAF was detected (Table 1). The treatment with TKI and panobinostat was applied at the cells derived from his resected tumor after they have been kept proliferating in in vitro culture conditions.

#### 3.4.1. Effects on Cell Viability of the Individual Tumor Cells Caused by Treatment with Sorafenib, Panobinostat and Selumetinib

**Figure 7:**
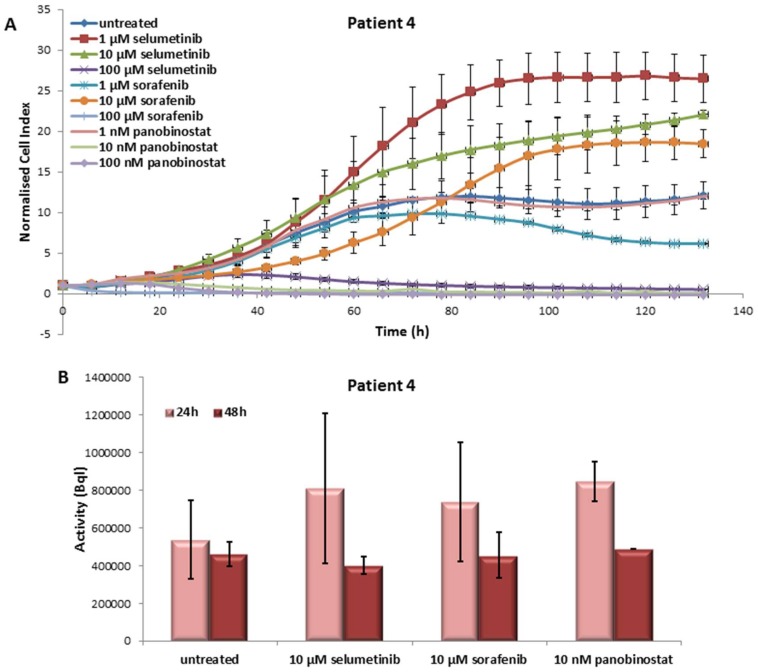
Effects of selumetinib, sorafenib and panobinostat on cell viability and RAI-U. Real-time cell viability of PDTT (PDTC—Patient 4) cells treated with 1 to 100 µM selumetinib and sorafenib and 1 to 100 nM panobinostat (**A**). RAI-U after 24 and 48 h of treatment with 10 µM selumetinib, 10 µM sorafenib and 10 nM panobinostat (**B**). Shown are means of experiments performed in triplicates ± SD (**A**)/SEM (**B**).

Selumetinib and sorafenib treatment caused a slight reduction of PDTT cell viability at 10 µM (Figure 7A). The highest concentration (100 µM) determined a significant reduction of the growth curve with values near to zero. Panobinostat showed a significant effect with 10 and 100 nM. These low concentrations reduced the cell viability down to zero.

#### 3.4.2. Radioiodine Up-Take after Treatment with Sorafenib, Panobinostat and Selumetinib

The function of NIS protein was monitored by RAI-U measurement after administration of the tree different compounds. RAI-U was not significantly up-regulated after 24 h of treatment with 10 µM selumetinib/10 µM sorafenib/10 nM panobinostat (Figure 7B). After 48 h of treatment, no variation of the radioiodine up-take was observed.

#### 3.4.3. Expression of *SLC5A5* (*NIS*), *HMGA2* and miRNAs

**Figure 8:**
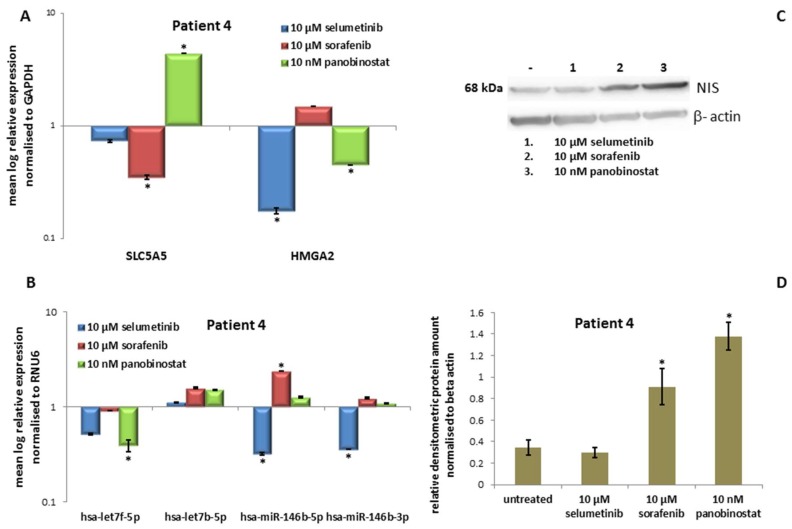
Effects of selumetinib, sorafenib and panobinostat on mRNAs, miRNAs and NIS protein expression in PDTT of patient 4 (PDTC). RT-qPCR of *NIS*, *HMGA2* (**A**), hsa-let7f-5p, hsa-let7b-5p, hsa-miR-146b-5p and hsa-miR-146b-3p (**B**). The expression was normalised to GAPDH (**A**) and RNU6 (**B**) set at 1.0. Shown are means of experiments performed in triplicates ± SEM. * *p* < 0.05 regarded as significant. Western blot detection of NIS protein in PDTT cells treated with 10 µM selumetinib, 10 µM sorafenib and 10 nM panobinostat (**C**). Beta-actin was detected as quality loading control; (**D**) densitometry of NIS protein level normalised to beta-actin. Shown are means of experiments performed in triplicates ± SEM. * *p* < 0.05 regarded as significant.

Treatment with 10 nM panobinostat induced a significant over-expression of *SLC5A5* transcript (Figure 8A). In addition, 10 µM sorafenib down-regulated the *SLC5A5* transcript level significantly, selumetinib treatment caused no change in the basal expression of *SLC5A5*. Treatment with 10 µM selumetinib and 10 nM panobinostat caused a significant down-regulation of *HMGA2* (Figure 8A). After treatment with 10 µM sorafenib, *HMGA2* transcript level was stable. Interestingly, the PDTT derived from this patient was also characterised by a significant down-regulation of hsa-let7f-5p after treatment with 10 nM panobinostat (Figure 8B). Treatment with 10 µM selumetinib caused a significant down-regulation of hsa-miR-146b-5p and hsa-miR-146b-3p. In addition, 10 µM Sorafenib induced an over-expression of hsa-miR-146b-5p.

#### 3.4.4. Expression of *NIS* Protein Level

Additionally, *NIS* protein level was analysed after 48 h of treatment with 10 µM selumetinib, 10 µM sorafenib and 10 nM panobinostat. As shown in Figure 8C, the treatment with 10 µM selumetinib did not cause any change in *NIS* protein level in comparison with untreated cells. In addition, 10 µM sorafenib and 10 nM panobinostat, especially caused a significant increase of *NIS* protein, further confirmed by densitometry (Figure 8D).

Here, panobinostat showed the highest efficacy with the lowest dosage (10 and 100 nM). Furthermore, panobinostat alone caused an over-expression of *NIS* transcript, protein level and activity (24 h) together with the suppression of *HMGA2* and hsa-let7f-5p. In addition, 10 µM selumetinib was able to down-regulate *HMGA2*, hsa-miR-146b-5p and hsa-miR-146b-3p and increase the radioiodine up-take (24 h). Furthermore, 10 µM sorafenib showed cytotoxic effects and increased NIS activity (24 h).

### 3.5. In Vitro Results of the Fifth Patient

The fifth patient was affected by PDTC and the overall survival was 174 weeks after his first diagnosis. The patient received once radiotherapy. Afterwards, the patient was treated with sorafenib and then with lenvatinib. Both treatments were interrupted because of severe adverse events. RAI-R was diagnosed. No mutation of BRAF was detected (Table 1). The treatment with TKIs and panobinostat was applied at the cells derived from his resected tumor after they have been kept proliferating in in vitro culture conditions.

#### 3.5.1. Effects on Cell Viability of the Individual Tumor Cells Caused by Treatment with Sorafenib, Panobinostat and Selumetinib

**Figure 9:**
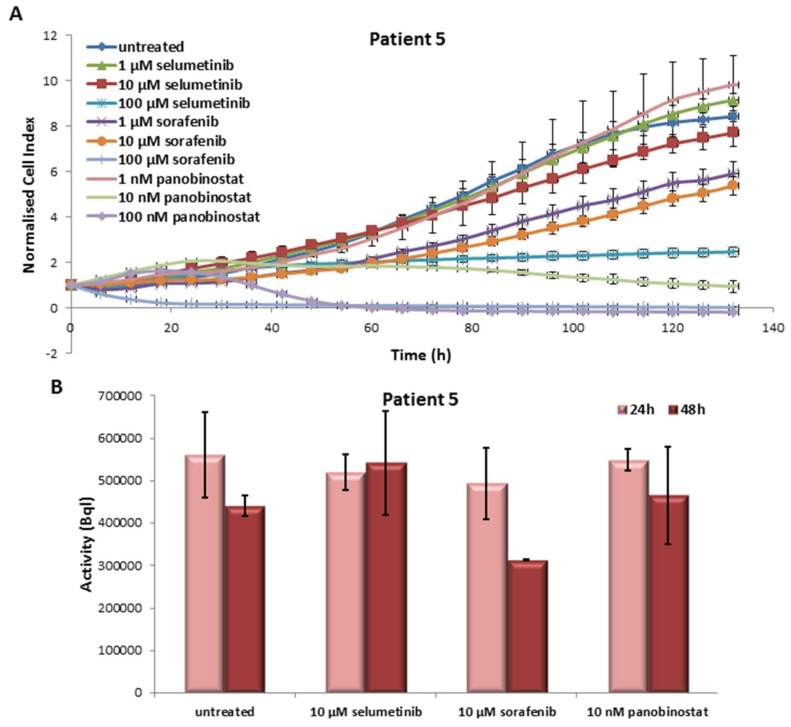
Effects of selumetinib, sorafenib and panobinostat on cell viability and RAI-U. Real time cell viability of PDTT cells (PDTC—Patient 5) treated with 1 to 100 µM selumetinib and sorafenib and 1 to 100 nM panobinostat (**A**). RAI-U after 24 and 48 h of treatment with 10 µM selumetinib, 10 µM sorafenib and 10 nM panobinostat (**B**). Shown are means of experiments performed in triplicates ± SD (**A**)/SEM (**B**).

In the PDTT cells of the fifth patient case, selumetinib caused a reduction of cell viability at 100 µM only. Treatment with sorafenib was able to reduce significantly the cell viability at 1 µM already. In addition, 10 and 100 nM panobinostat showed, once again, the strongest efficacy in cell viability reduction (Figure 9A).

#### 3.5.2. Radioiodine Up-Take after Treatment with Sorafenib, Panobinostat and Selumetinib

As shown in Figure 9B, the treatment with the three compounds showed an unchanged radioiodine up-take after 24 h and 48 h in comparison with untreated cells.

#### 3.5.3. Expression of *SLC5A5* (*NIS*), *HMGA2* and miRNAs

**Figure 10:**
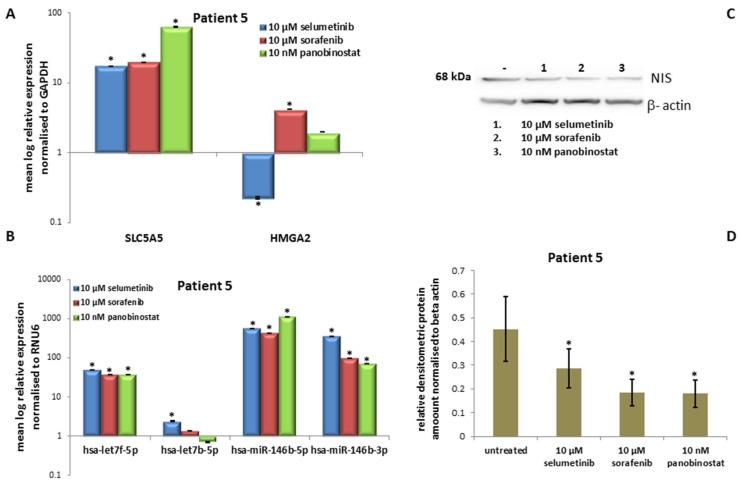
Effects of selumetinib, sorafenib and panobinostat on mRNAs, miRNAs and NIS protein expression in PDTT of patient 5 (PDTC). RT-qPCR of *NIS*, *HMGA2* (**A**), hsa-let7f-5p, hsa-let7b-5p, hsa-miR-146b-5p and hsa-miR-146b-3p (**B**). The expression was normalized to GAPDH (**A**) and RNU6 (**B**) set at 1.0. Shown are means of experiments performed in triplicates ± SEM. * *p* < 0.05 regarded as significant. Western blot detection of NIS protein in PDTT cells treated with 10 µM selumetinib, 10 µM sorafenib and 10 nM panobinostat (**C**). Beta-actin was detected as quality loading control; (**D**) densitometry of NIS protein level normalized to beta-actin. Shown are means of experiments performed in triplicates ± SEM. * *p* < 0.05 regarded as significant.

All three compounds were able to cause an over-expression of *NIS* transcript (Figure 10A). Sorafenib and panobinostat caused an over-expression of *HMGA2* transcript, whereas selumetinib down-regulated its expression (Figure 10A).

The up-regulation of hsa-let7f-5p, hsa-miR-146b-5p and hsa-miR-146b-3p was observed after treatment with 10 µM selumetinib, 10 µM sorafenib and 10 nM panobinostat. Hsa-let7b-5p showed a stable expression after treatment (Figure 10B).

#### 3.5.4. Expression of NIS Protein Level

NIS protein level was significantly reduced after treatment with TKIs and HDACI (Figure 10C) and confirmed by densitometry analysis (Figure 10D).

Here, it could be observed a significant cytotoxic effect exerted by treatment with sorafenib and panobinostat. All three compounds were able to increase the expression of *NIS* transcript.

However, no increase of RAI-U was observed and NIS protein level decreased after treatment with the three compounds. The miRNAs were up-regulated after treatment, thus could not be inversely correlated with *NIS* over-expression. Additionally, the onco-miRNAs 146b were over-expressed after treatment with the three compounds.

### 3.6. Human Foreskin Fibroblast

#### 3.6.1. Effects on Cell Viability of Human Fibroblast Caused by Treatment with Sorafenib, Panobinostat and Selumetinib

**Figure 11:**
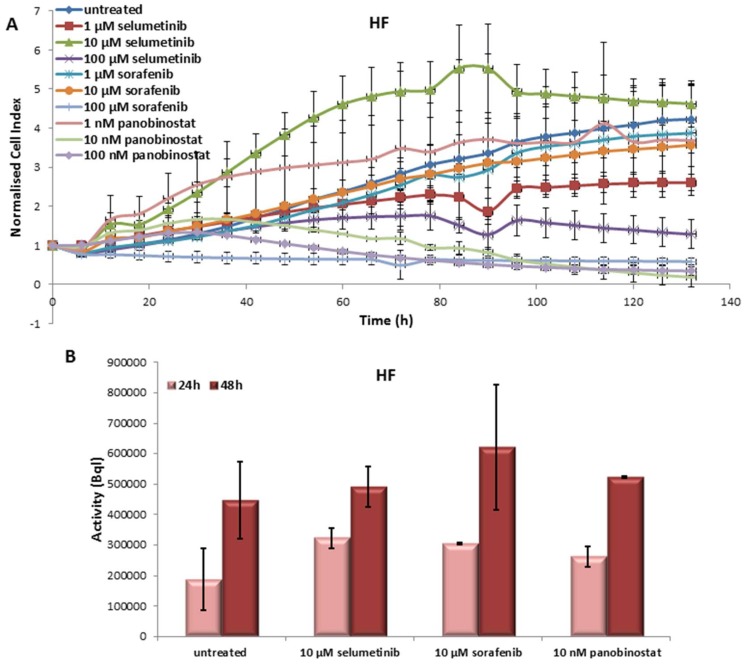
Effects of selumetinib, sorafenib and panobinostat on cell viability and RAI-U. Real-time cell viability of human foreskin fibroblasts (HF) cells treated with 1 to 100 µM selumetinib and sorafenib and 1 to 100 nM panobinostat (**A**). RAI-U after 24 and 48 h of treatment with 10 µM selumetinib, 10 µM sorafenib and 10 nM panobinostat (**B**). Shown are means of experiments performed in triplicates ± SD (**A**)/SEM (**B**).

Human fibroblasts (HF), used as control cells, were affected, in general, by treatment with TKIs and HDACI. In particular, cell viability was reduced by 1 to 100 µM selumetinib, 100 µM sorafenib and 10 to 100 nM panobinostat (Figure 11A).

#### 3.6.2. Radioiodine Up-Take after Treatment with Sorafenib, Panobinostat and Selumetinib

HF treated with 10 µM selumetinib, 10 µM sorafenib and 10 nM panobinostat showed an increase of RAI-U after 24 and 48 h (Figure 11B).

#### 3.6.3. Expression of *SLC5A5* (*NIS*), *HMGA2* and miRNAs

**Figure 12:**
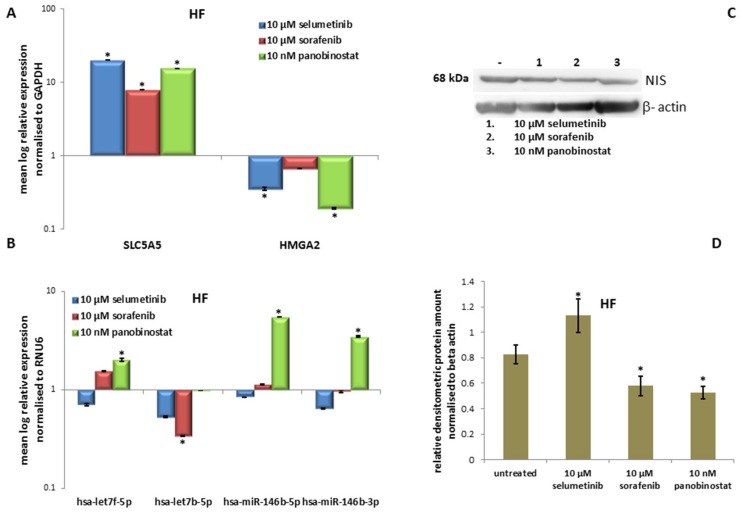
Effects of selumetinib, sorafenib and panobinostat on mRNAs, miRNAs and NIS protein expression in HF. RT-qPCR of *NIS*, *HMGA2* (**A**), hsa-let7f-5p, hsa-let7b-5p, hsa-miR-146b-5p and hsa-miR-146b-3p (**B**). The expression was normalised to GAPDH (**A**) and RNU6 (**B**) set at 1.0. Shown are means of experiments performed in triplicates ± SEM. * *p* < 0.05 regarded as significant. Western blot detection of NIS protein in HF cells treated with 10 µM selumetinib, 10 µM sorafenib and 10 nM panobinostat (**C**). Beta-actin was detected as quality loading control. (**D**) Densitometry of NIS protein level normalised to beta-actin. Shown are means of experiments performed in triplicates ± SEM. * *p* < 0.05 regarded as significant.

The transcript level of *NIS* was significantly over-expressed after treatment with the three compounds (Figure 12A). *HMGA2* was significantly down-regulated after treatment with 10 µM selumetinib and 10 nM panobinostat (Figure 12A). Furthermore, it could be observed that 48 h treatment with panobinostat induced the over-expression of has-let7f-5p, has-miR-146b-5p and hsa-miR-146b-3p. In addition, 10 µM sorafenib reduced significantly the expression of hsa-let7b-5p (Figure 12B).

#### 3.6.4. Expression of NIS Protein Level

The protein level of NIS was significantly up-regulated after treatment with 10 µM selumetinib and stable after treatment with 10 µM sorafenib and with 10 nM panobinostat (Figure 12C). The protein expression was analysed densitometrically (Figure 12D). 

The human fibroblast, used as control, showed a reduction of cell viability mediated by TKIs and panobinostat. Additionally, these cells had an increase of RAI-U independently of the administered compound together with the over-expression of NIS transcript. Even HMGA2 was down-regulated after treatment with 10 µM selumetinib and 10 nM panobinostat. MiRNAs were generally stable or up-regulated after treatment, except for hsa-let7b-5p that was down-regulated by 10 µM sorafenib.

We could find evidence that the effect of different compounds had a similar effect in normal cells used as control for the proposed study.

## 4. Discussion

This study first focused on the evaluation of variable responses of PDTTs derived from PDTCs and ATCs to the treatment with two different TKIs, selumetinib and sorafenib, and one HDACI, panobinostat. The study focused on the expression of *NIS*, *HMGA2*, associated miRNAs and RAI-U modulation after treatment.

Our findings revealed that every PDTT enrolled in the study showed variable individual sensitivity to the treatment with TKIs and HDACI.

Here, we could confirm—to our knowledge for the first time—the high potency of panobinostat in comparison with TKIs for the treatment of ATCs—and PDTCs-derived PDTT cells. This finding also confirms previous studies showing panobinostat as an effective treatment option for aggressive thyroid cancer [26,27]. In particular, panobinostat showed the strongest cytotoxic effect in all PDTTs independently of their tumor type. Except for the first patient-derived tumor tissue culture, panobinostat showed already an overall cytotoxic effect at 10 nM. The response to sorafenib and selumetinib was quite different in all PDTT cells. Their cytotoxic effects were detected only at higher concentrations than panobinostat. In addition, 100 µM sorafenib was cytotoxic in all PDTTs, whereas 100 µM selumetinib caused a reduction of cell viability in the PDTT cells of patient 2, 3 and 4. Interestingly, the three compounds caused a significant reduction of cell viability in human fibroblasts used as control.

It is already known that treatment with panobinostat may lead to *NIS* RNA and protein expression and to a relevant (125) radioiodine up-take in ATC [28,29]. Hong et al. even stated that HDACIs can induce the RAI-U in patients affected by RAI-R thyroid cancer [30].

In our study, all PDTT cells showed a strong significant over-expression of *NIS* transcript after treatment with panobinostat. In one PDTC (patient 5) only, all three compounds induced an up-regulation of *NIS.* In all the other cases neither selumetinib nor sorafenib were able to up-regulate the *NIS* transcript.

The different sensitivity of the PDTT cells to the treatment with different compounds highlights the importance of using primary tumor cells to individualise the most efficacious therapy for patients with these thyroid cancers and to exclude the ineffective compounds. Moreover, this study revealed the poor efficacy of TKIs in terms of *NIS* restoration/over-expression at transcript level. Furthermore, the function of NIS protein was analysed by RAI-U measurement after administration of the three different compounds. The present study could confirm that the treatment with selumetinib, sorafenib and panobinostat was able to increase the RAI-U in thyroid cancer cells as previously shown [31]. However, we could observe quite different reactions—three PDTTs (patients 1, 2 (ATC) and 4 (PDTC)) showed an increased RAI-U after treatment with all three compounds, one PDTC (patient 5) showed no RAI-U and one ATC-derived PDTT (patient 3) showed a significant up-regulation of NIS activity after treatment with 10 µM selumetinib. In contrast with the finding of Ho et al. that BRAFV600E-mutated cell lines show a better RAI-U after the treatment with selumetinib [32], it could be here observed that the RAI-U was not correlated with the mutation status of BRAFV600E of the PDTT cancer cells.

As previously reported, NIS expression could be modulated by the expression of miRNAs belonging to Let7 family. In particular, NIS transcript in silico analysis revealed NIS as validated target of hsa-let7f-5p [23]. Four PDTTs (patients 1, 2, 3 and 4) showed a down-regulation of hsa-let7f-5p after treatment with panobinostat; only one PDTC (patient 5) did not show any change. As previously shown, hsa-let7f-5p was found to be up-regulated in all histopathological subgroups of TC [17]. The possibility to suppress its expression in order to restore *NIS* transcript could represent a valuable target for the treatment of ATC and PDTC.

The stable/up-regulated expression of hsa-let7b-5p after treatment could be inversely correlated with the down-regulation of *HMGA2*, a non-transcription factor with well-known oncogenic properties as previously shown in liver cancer cell lines [25]. Thus, showing that the treatment with TKIs and HDACI can re-establish an inverse correlation between hsa-let7b-5p and HMGA2, which was found to be absent in ATC samples of patients collected in the previous study [23].

The oncogene *HMGA2* was stably expressed or down-regulated after the treatment with panobinostat, selumetinib and sorafenib in all cases included in the study. Only in one case of PDTC (patient 5), sorafenib and panobinostat induced an up-regulation of HMGA2.

The hsa-miR-146b-5p and hsa-mir-146b-3p are over-expressed in several malignancies including thyroid cancer. They are responsible for promoting epithelial-mesenchymal transition and metastasis in PTC [12,18,19,20]. These miRNAs were found not expressed in ATC [32].

Interestingly, in our study, it could be found that these miRNAs were stable and or up-regulated in four PDTTs (patients 1, 2, 3 and 5) derived from ATC and PDTC, whereas patient 4 (PDTT derived from PDTC) showed a down-regulation of both hsa-miR-146b-5p and hsa-miR-146b-3p after treatment with 10 µM selumetinib. It becomes clear that these miRNAs could play a key role in the cell death mechanisms induced by treatment with TKIs and panobinostat. Further investigations are needed to clarify the mechanisms regulated by hsa-miR-146b in ATC and PDTC.

Finally, NIS protein level was found regulated by treatment with selumetinib, sorafenib and panobinostat. In particular, patient 1-derived PDTT cells, which have no basal level of NIS, showed an over-expression of it after treatment with 10 µM selumetinib, 10 µM sorafenib and 10 nM panobinostat. Furthermore, patients 3 and 4 PDTT cells showed an over-expression of NIS protein after treatment with 10 µM sorafenib and 10 nM panobinostat. The level of NIS protein was down-regulated after treatment with the three compounds in PDTT from patients 2 and 5.

In terms of correlation between *NIS* transcript expression, radioiodine up-take and expression of NIS protein after treatment with TKI and panobinostat, it could be found a discrepancy between all PDTT used in the study. Even though *NIS* transcript was generally up-regulated after treatment, its activity in terms of radioiodine up-take and its protein level were differently modulated. Only the PDTTs derived from patients 1 and 4 showed an up-regulation of *NIS* transcript, activity and protein level after treatment, especially, with 10 nM panobinostat. The other patients showed a stable radioiodine up-take or even a suppression of NIS protein level that could not be functionally correlated with the significant over-expression of NIS transcript. Here, we could only hypothesize that some patients could express a non-functional/distorted NIS protein, as previously reported [10]; and/or that NIS protein membrane localization and activity is dependent on its glycosylation status as previously described in thyroid cancer [10]. Further studies should focus on the activity of NIS promoter as it has been previously shown in breast cancer cells [33]. Additionally, the post-translational modifications occurring at NIS protein should be further investigated to better understand its function and its subcellular localization. 

Nevertheless, there are some limitations referring the method of analysing PDTT of ATCs/PDTCs. Concerning the clinical course of the patients enrolled in this study, it has been observed that the patients suffering from ATC are mainly older people with multiple comorbidities. Furthermore, it is well known that ATCs are very aggressive tumors with early invasion of surrounding tissue. To our knowledge, the procedure of preparing PDTT including the testing of different compounds takes about six weeks—this might be a long time for patients suffering of ATC; patients 1 and 2 died after less than six weeks. Moreover, it is a time- and money-consuming method to prepare PDTT. In our opinion, it is a promising method for selected patients suffering from ATCs and especially PDTCs with no more treatment options. Considering the clinical course of the patients suffering from PDTC, they obviously need individual and personalised treatment options suitable for their individual tumors. Additionally concerning the PDTCs, they might change their tumor behaviour during the progression of the disease and develop resistances after radioiodine therapy, which might require alternative treatment options. Looking at patient 5, suffering from PDTC, sorafenib was an effective drug at the beginning, but, in the further clinical course, an elevation of the tumor marker thyroglobuline (TG) was recorded, thus providing evidence of a tumor recurrence. An additional treatment with lenvatinib caused again a decline of TG.

## 5. Conclusions

In conclusion, a successful treatment strategy might be on in vitro testing of patient-derived tumor tissue, since every ATC- or PDTC-derived PDTT shows different response to the treatment with certain HDACIs and/or TKIs. Considering the clinical course of the patients described in this study, the treatment with TKIs and HDACIs might be associated with multiple adverse events, which might require a dose reduction or even the stop of a therapy. Patient 3, for example, needed a dose reduction and furthermore caused even the termination of the therapy with sorafenib due to increasing polyneuropathy. In this case, the need of alternative treatment options—for example in terms of HDACIs—would be required. Especially, HDACI treatment of ATC/PDTC seems to be promising, thus leading to a better prognosis in patients affected by these rare malignancies.

## Figures and Tables

**Table 1:** ATC patients characterisation based on clinical data collected from tumor board and histopathological analysis.

Patient	1	2	3
Date of diagnosis (month/year)	April/2015	December/2016	September/2012
Gender	Male	Male	Male
Age at diagnosis (years)	83	81	42
Histopathology ^1^	ATC	ATC	ATC
Tumor origin	Thyroid tissue	Thyroid tissue	Thyroid tissue
Tumor stage at diagnosis ^2^	UICC Stage: IVC on the right side	UICC Stage: IVB	UICC Stage: IVB
TNM-stage	pT4a pN1a (21/32) pM1	pT4b pN1b (2/5) pM0	pT4b pN0 (0/36) pM0
Mutation-status	BRAFV600E+	BRAFV600E−	BRAFV600E−
Aerodigestive infiltration	Trachea	-	-
Primary surgery	Thyroidectomy + bilateral cervico-central + unilateral cervico-lateral lymphadenectomy	Hemi-thyroidectomy + cervicocentral lymphadenectomy+ resection of the recurrent laryngeal nerve on one side	Thyroidectomy + bilateral cervicocentral + cervicolateral lymphadenectomy
Resection status ^3^	R2	R2	R0
External beam radiation	-	-	Yes
Chemotherapy	-	-	Docetaxel + Cisplatin
Radioiodine therapy	-	-	-
Follow–up	-	-	June/2013: pulmonary metastasis -> video-assisted thoracoscopic wedge resection
Individual treatment design	No	No	Sorafenib -> adverse events: polyneuropathy, pain in muscles and bones
Palliative treatment	Yes	Yes	-
Overall survival after diagnosis (weeks)	2	8	282

^1^ ATC = anaplastic thyroid carcinoma, PDTC = poorly differentiated thyroid carcinoma; ^2^ UICC (Union for International Cancer Control) 7th edition 2009; ^3^ R0 = microscopically complete resection, R1 = microscopically incomplete resection, R2 = macroscopically incomplete resection. TNM stage: Tumor Nodes Metastasis Classification of Malignant Tumor.

**Table 2:** PDTC patients characterization based on clinical data collected from tumor board and histopathological analysis.

Patient	4	5
Date of diagnosis (month/year)	May/2010	November/2012
Gender	Male	Female
Age at diagnosis (years)	73	44
Histopathology ^1^	PDTC	PDTC
Tumor origin	Lymph node metastasis	Lymph node metastasis
Tumor stage at diagnosis ^2^	UICC Stage: I	UICC Stage: I
TNM-stage	pT2 pN0 pM0	pT3 pN0 (0/19) pM0
Mutation-status	BRAFV600E−	BRAFV600E−
Aerodigestive infiltration	-	-
Primary surgery	Thyroidectomy + bilateral cervico-central lymphadenectomy	Thyroidectomy + bilateral cervicocentral + cervicolateral lymphadenectomy
Resection status ^3^	R0	R1
External beam radiation	-	Yes
Chemotherapy	-	Docetaxel + Cisplatin
Radioiodine therapy	Yes (three times between 2010 and 2011)	Yes (one time in 2012)
Follow–up	September/2011: cervical, osseus and pulmonary metastases -> treatment with sorafenib -> due to adverse events chemotherapy with carboplatin + etoposid September/2014: reexploration of the neck with tumordebulking to establish primary cell culture -> again therapy with sorafenib	November/2013: bone and pulmonary metastases -> surgical resection July/2014: osseus, hepatic and pulmonary progress -> re-exploration of the neck with resection of cervical lymphnode metastases to establish primary cell cultures October/2014: progress of metastases -> treatment with sorafenib April/2015: progress of metastases -> treatment with Lenvatinib
Individual treatment design	Sorafenib -> adverse events: polyneuropathy, dry skin, sore throat	Sorafenib -> adverse events: polyneuropathy, diarrhea, sore throat Lenvatinib -> adverse events
Palliative treatment	-	Yes (since September/2015)
Overall survival after diagnosis (weeks)	396	174

^1^ ATC = anaplastic thyroid carcinoma, PDTC= poorly differentiated thyroid carcinoma; ^2^ UICC (Union for International Cancer Control) 7th edition 2009; ^3^ R0 = microscopically complete resection, R = microscopically incomplete resection, R2 = macroscopically incomplete resection. TNM stage: Tumor Nodes Metastasis Classification of Malignant Tumor.
